# Nitric oxide delays yellowing by regulating chloroplast membrane lipid metabolism and the ascorbate–glutathione cycle in postharvest broccolini

**DOI:** 10.3389/fnut.2026.1785583

**Published:** 2026-02-23

**Authors:** Wenzheng Gao, Chen Li, Guobao Niu, Yonghong Ge, Feng Luo

**Affiliations:** 1 Liaoning Provincial Professional Technology Innovation Center of Meat Processing and Quality-Safety Control, College of Food and Health, Jinzhou Medical University, Jinzhou, China; 2 State Key Laboratory of Vegetable Bio-Breeding, Tianjin Academy of Agricultural Sciences, Tianjin, China; 3 College of Food Science and Engineering, Bohai University, Jinzhou, China

**Keywords:** antioxidation, broccolini, color, membrane lipid, nitric oxide, spanning protein

## Abstract

**Introduction:**

NO plays an important role in regulating fruit and vegetable postharvest quality. However, its mechanisms of action in delaying postharvest yellowing of vegetables remains poorly understood.

**Methods:**

To elucidate this, we examined the effects of an NO scavenger (cPTIO), and an exogenous NO donor (GSNO), on broccolini postharvest yellowing. Furthermore, the mechanism by which NO delays the postharvest chlorosis process of broccolini was revealed through the membrane lipid metabolism and ascorbate-glutathione cycle pathways.

**Results:**

Evaluation of color and chlorophyll content revealed that GSNO treatment delayed yellowing by 4 days relative to the control. The chloroplast membrane remained well developed during the entire storage process following GSNO treatment, whereas it began to collapse 4 days after cPTIO treatment. GSNO maintained cell-membrane permeability, increased levels of chloroplast membrane lipids (monoglycosyldiacylglycerol, phosphatidylglycerol, and digalactosyldiacylglycerol), improved *ω*-3 FAD enzyme activity, enhanced ascorbate–glutathione cycling, and promoted *BoFAD8* transcription, thus promoting 18/16:3-triene fatty acid accumulation. GSNO upregulated *BoPPT1*, which was identified as a chloroplast membrane-spanning protein that participates in regulating chloroplast morphology.

**Discussion:**

NO alleviated oxidative damage to the chloroplast membrane lipid system, thereby delaying broccolini yellowing. The results of this study may provide novel insights for developing postharvest quality control technologies for broccolini and the genetic improvement of broccolini varieties.

## 1 Introduction

Membrane lipid metabolism plays a core role in the postharvest senescence of plants. After postharvest organs (such as leaves, fruits, and flowers) are detached from the mother plant, they lose water and nutrient supply, and metabolic imbalance accelerates senescence. The dynamic changes in membrane lipid metabolism directly affect membrane fluidity, permeability, signal transduction, and the stability of organelles, thereby regulating postharvest senescence (1). The integrity and functional state of the cell membrane system are one of the key factors determining the rate of senescence. In *Pteridium aquilinum* var. *latiusculum*, 1-MCP treatment effectively delayed the reduction of unsaturated fatty acid content and the unsaturated fatty acid/saturated fatty acid ratio in cell membranes, thereby extending the shelf life (2). The treatment of burdock fructooligosaccharide could effectively delay the sharp decline of unsaturated fatty acid content in blueberry fruits during the shelf life, weaken the membrane lipid metabolism process, maintain the integrity of the cell membrane, and thus delay senescence (3). The results of lipid metabolism indicated that the composition form of phosphatidylcholine 34:2/36:2/36:4, phosphatidylethanolamine 34:2, and diacylglycerol 36:4 directly affects the postharvest storage aging of Soybean (4). Similar results have also been confirmed in and *indica* (Fengliangyou) and *japonica* (Nanjing 46) rice during the post-harvest storage period (5).

Postharvest loss of quality in vegetables is mostly caused by reactions between free radicals and proteins, enzymes, polyunsaturated fatty acids, and other macromolecules that are converted into active peroxides (6, 7). Non-enzymatic antioxidant scavenging systems (comprising ascorbate [AsA] and glutathione [GSH], which are formed by the polymerization of glutamic acid, cysteine, and glycine), are key pathways for reactive oxygen species (ROS) scavenging within cells. They eliminate ROS via degradation, providing resistance toward oxidative damage induced by abiotic stress. Excess cellular H_2_O_2_ is cleared primarily via the AsA–GSH pathway (8, 9), which includes glutathione reductase (GR), ascorbate peroxidase (APX), monodehydroascorbate reductase (MDHAR), and dehydroascorbate reductase (DHAR). Alternatively, H_2_O_2_ can be eliminated by oxidizing GSH to produce oxidized glutathione. Dysregulation of ROS elimination in plants leads to toxic effects, especially membrane peroxidation, damaging or breaking down the membrane system that maintains cell regionalization and damaging the biofilm system, leading to poor-quality fruits and vegetables after harvest (10).

Chloroplasts, mitochondria, and microsomes exhibit high levels of oxidative activity and strong electron transfer functions, and chloroplasts are the main source of ROS in green plant tissues. Chloroplasts, bilayer organelles, are plastids occurring mainly in the leaves or other green parts of plants; they are located in the area between the large vacuole in the center of the cell and cell wall (11, 12). Most chloroplasts are fusiform, comprising the stroma, a chloroplast envelope, and thylakoids; they exhibit three membranes (outer, inner, and thylakoid membranes) and three lumens (intermembrane space, stroma, and thylakoid lumen) (13). Chloroplasts play a crucial role in chlorophyll (Chl) storage. Various factors can induce changes in their chemical composition, morphology, and structure, thus altering their function. Changes in chloroplast structural integrity and morphology affect Chl content, directly affecting fruit and vegetable yellowing (14). Yellowing can be induced by factors including nutritional stress, pathogenic infection, hormonal balance, water loss, temperature fluctuations, energy consumption, metal ion imbalance, and cell decay. Nonetheless, the intrinsic relationships between chloroplast membrane lipid metabolism and postharvest yellowing in vegetables remain poorly understood.

NO is an important component of NO-dependent signaling pathways. Endogenous nitric oxide (NO) is derived primarily from an exogenous NO donor, *S*-nitrosoglutathione (GSNO). cPTIO [2-(4-carboxyphenyl)-4,4,5,5-tetramethylimidazoline-1-oxyl-3-oxide], an NO scavenger, reacts with NO to generate carboxyl-PTI derivatives and simultaneously produce nitrite or nitrate, thereby participating in NO elimination (15, 16). As a gas signal effector, NO actively participates in ethylene biosynthesis; hormone crosstalk among multiple plant hormones; glucose, polyamine, cell-wall, and energy metabolism; antioxidant system functions; and *S*-nitrosylation. It thus plays an important role in regulating the postharvest quality of fruits and vegetables, by delaying ripening and aging, alleviating cold damage, reducing browning incidence, and inhibiting the occurrence of postharvest diseases (17). In sweet cherries, GSNO treatment significantly improves AsA–GSH cycling and reduces the ROS content, thus delaying quality loss during cold storage (18). In citrus, exogenous NO treatment can induce peroxidase (POD) and the AsA–GSH cycle, enhancing their resistance to *Colletotrichum gloeosporioides* and prolonging their postharvest storage (19). However, fruits and vegetables respond differently to NO, and the mechanisms whereby postharvest yellowing is delayed remain unclear.

The tender florets of broccolini (*Brassica oleracea* L. var. *botrytis* × *B. alboglabra*) are primarily consumed as food, and have a beautiful green color and sweet taste. Broccolini is rich in anti-cancer ingredients, more so than broccoli and kale. It contains various bioactive ingredients, including vitamins, minerals, and proteins, and is considered a high-quality and high-end functional green vegetable that combines excellent flavor and health benefits (20). Harvested broccolini loses a large amount of nutrients under unsuitable external conditions, and the curd composed of buds is prone to wilting, dehydration, and even yellowing, thereby reducing its value and severely limiting its sales. We hypothesized that NO treatment improves antioxidant protection ability and regulates chloroplast membrane metabolism, thereby improving postharvest storage quality in broccolini. To elucidate these mechanisms, we determine the effects of inducing the chloroplast membrane lipid metabolism and AsA–GSH cycle in broccolini via exogenous NO treatment. These findings may support future research into the quality of broccolini during postharvest storage and logistics, and may enhance the economic benefits of cruciferous crops.

## 2 Materials and methods

### 2.1 Plant materials and treatment

‘Qianxiu’ broccolini was obtained from the Tianjin Academy of Agricultural Sciences, China (116°E, 39°N). Picking standards require broccolini to grow consistently, with no pests, diseases, or damage to the flower bud surface. Every 100 broccolini were treated with 150 μmol L^−1^ cPTIO or 80 μmol L^−1^ GSNO; these optimal doses were determined via preliminary experiments. The control group samples were immersed in distilled water. After treatment, the samples were soaked for 15 min, then dried in the shade before being packaged in 0.02 mm polyethylene bags. The packaged samples were stored in a constant-humidity and -temperature cabinet, at 80% relative humidity and 20 °C. All experiments were conducted every 2 days until the end of the period of severe yellowing. Subsequently, the curd buds were frozen in liquid nitrogen and stored in an ultra-low temperature refrigerator for future determination of enzyme activity and gene expression. Other experiments were conducted using fresh samples.

### 2.2 Determination of color parameters

The data were measured according to a five-point sampling method using a Chroma Meter grating spectrophotometer (YS3060; Sanen Time Intelligent Technology Co., Ltd., Guangdong, China). The color parameters included b* (yellowness), a* (greenness), and L* (lightness). The yellowing index (YI) and the color difference (Δ*E*) were calculated as follows Equations (1, 2):


$$\mathrm{YI}=(b^{*}/L^{*})\times 142.86$$ (1)

$$\mathrm{\Delta E}={\left[{\left(\Delta b^{*}\right)}^{2}+{\left(\Delta L^{*}\right)}^{2}+{\left(\Delta a^{*}\right)}^{2}\right]}^{1/2}$$ (2)

### 2.3 Ultrastructural observations

Sections were prepared using an EMUC6 Ultra-Thin Micrograph (Leica Biosystems, Wetzlar, Germany), stained with uranyl acetate solutions and lead citrate, and subjected to transmission electron microscopy (JEM1400; Japan Electronics Co., Ltd., Tokyo, Japan) to observe cell structure (14).

### 2.4 Determination of chlorophyll content

A liquid chromatograph mass spectrometer (LC–MS 2020; Shimadzu, Kyoto, Japan) with a C18 chromatographic column (Slum-pack GIS, 2 μm × 75 mm × 2.1 mm; Shimadzu) was used; the injection parameters were set at 30 μL injection volume and 1.3 mL min^−1^ flow rate at 40 °C. Solutions A [water: methanol: 0.05 mol L^−1^ ammonium acetate = 1:8:1] and B (acetone: methanol = 1:1) formed the mobile-phase solvent system (with volume-ratio mixing). The program was set to elute along a gradient. Initially, 75% A and 25% B were used to elute for 5 min, followed by 25% A for 8 min, 10% A for 10 min, 100% B for 5 min, and, finally, 75% A and 25% B for 7 min. After the column was balanced, Chl *a* and *b* standards were used to construct standard curves for quantitative analysis (21).

### 2.5 Determination of relative conductivity, malondialdehyde (MDA) and H_2_O_2_ content

#### 2.5.1 Relative conductivity

The buds from each group were removed, placed in a beaker, rinsed five times with deionized water, and dried using filter paper to absorb excess water. Thereafter, the samples were soaked in deionized water at 20 °C for 4 h, after which conductivity (*C*_0_) was measured using a BEC-6800 Conductivity Meter (BELL Analytical Instruments Co., Ltd., Dalian, China). Subsequently, the samples were boiled for 20 min, cooled to 20 °C, and the solution conductivity (*C*) was measured again (22). Relative conductivity (as %) was calculated as relative conductivity = (*C*_0_/*C*) × 100.

#### 2.5.2 MDA content

5 mL 10% trichloroacetic acid solution was added to a 1 g tissue sample and centrifuged at 10,000 × *g* for 25 min to obtain the supernatant. Next, 2 mL of 0.67% 2-thiobarbituric acid solution was added and mixed well before boiling in water for 20 min. When the temperature was lowered to 20 °C, the mixture was centrifuged at 10,000 × *g* for 20 min, after which absorbance values were measured at 600, 450, and 532 nm. The MDA content was calculated as follow: [6.45 ×(OD_532_ – OD_600_) – 0.56 × OD_450_] × *Vt*/(*Vs* × *m*), where *Vt* represents the total volume of the extraction solution, *Vs* the volume of the extraction solution taken during the determination, and *m* represents the mass of the sample (23).

#### 2.5.3 H_2_O_2_ content

Each 1.0 g tissue sample was ground in an ice bath with 2 mL acetone and centrifuged at 10,000 *× g* for 15 min. Thereafter, 400 μL of the supernatant was mixed with 120 μL of 20 mol L^−1^ ammonia water and 80 μL of 2% titanium sulfate, followed by centrifugation at 8,000 × *g* for 15 min at 20 °C. After centrifugation, 800 μL 2.5 mol L^−1^ H_2_SO_4_ was used to fully dissolve the precipitate, followed by centrifugation at 8,000 × *g* for 5 min at 20 °C. A Multiskan FC Microplate Reader (BELL Analytical Instruments Co., Ltd.) was used to measure absorbance at 415 nm, and an H_2_O_2_ concentration gradient standard curve was used to determine the H_2_O_2_ content of the sample (24).

### 2.6 Extraction and measurement of membrane lipids

Half-gram intact broccolini tissue samples were soaked in a 5 mL propan-2-ol solution containing 0.01% butylated hydroxytoluene (BHT) in a water bath at 75 °C for 10 min. A mixture of ultrapure water (0.6 mL) and chloroform (1.5 mL) was added to the above mixture for homogenization in the dark for 2 h, after which 4 mL of a chloroform–methanol mixture containing 0.01% BHT was added, shaken for 20 min, and the extract collected. This process was repeated until the sample was entirely eluted to colorless. Thereafter, 1 mL of KCL (1 M) was added, and after mixing, 5 mL of ultrapure water was added; the mixture was centrifuged at 800 × *g* for 10 min to remove the aqueous phase, and the samples were dried with nitrogen. Subsequently, the extracted buds were dried at 105 °C for 8 h and then weighed (25). Finally, the membrane lipid content of the samples was determined using a QTRAP 4500 High-Performance Liquid Chromatography Electrospray Ionization Tandem Mass Spectrometer (Bona Ajer Co., Ltd., Framingham, MA, USA).

### 2.7 Fatty acid component determination

Fifteen-gram samples were sterilized at 100 °C for 15 min and cooled to room temperature. Next, the samples were ground in 15 mL of a chloroform–methanol mixture and centrifuged at 8,000 × *g* for 15 min. Thereafter, 10 mL of 0.76% NaCl solution was added to the lower layer solution and shaken well. After layering, the lower layer extract was collected and dried to obtain the total lipids. Afterward, a mixture of methanol-saturated petroleum ether and petroleum ether-saturated methanol (1:1, by volume) was sequentially added to the total fat, followed by sequential addition of petroleum ether-saturated methanol and methanol-saturated petroleum ether mixture (6:1, by volume) after shaking and homogenization. The mixed solution was concentrated, and the polar fatty acids were dissolved in 1 mL of MeOH at room temperature for retention. Subsequently, 4 mL of a benzene petroleum ether mixed solution and 1 mL of 0.4 mol L^−1^ KOH solution were added to the sample and mixed well, followed by the addition of 10 mL of distilled water; the admixture was mixed and allowed to stand to obtain the upper solution, which was then dissolved in *n-*hexane. Furthermore, helium was used as the carrier gas in a 0.2 mm × 25 m × 0.33 μm TPA capillary column (Thermo Fisher Scientific, Waltham, MA, USA), and a TSQ-8000 Evo GC–MS (Thermo Fisher Scientific) was used to isolate and determine the fatty acid components. The program was set with 80 °C, with a 20:1 partition ratio and 1.5 mL min^−1^ flow rate (26).

### 2.8 AsA/Vitamin E (V_E_) content determination

AsA content was determined using an LC–MS 2020 (Shimadzu) at an injection flow rate of 1.0 mL min^−1^ and 15 μL injection volume. The samples were separated using a C18 chromatographic column (Slum pack GIS, 2 μm × 75 mm × 2.1 mm; Shimadzu). An equal elution program was set up, and the mobile phase system was prepared according to the material volume ratio (13:13:39:13) that included 2.5 mmol L^−1^ ammonium chloride, 1.25 mmol L^−1^ disodium dihydro EDTA, 2% acetonitrile, and 50 mmol L^−1^ NaH_2_PO_4_ (27). The quantitative AsA levels results are expressed in mg AsA/100 g FW.

*V_E_* content was determined using a 2475 Fluorescence Detector (Waters 2695 SPE-HPLC; Waters Corporation, Milford, MA, USA). Sample separation was conducted using a 4.6 mm × 250 mm Ultimate AQ-C18 chromatographic column (Waters Corporation). The injection volume, column temperature, and flow rate were 10 μL, 30 °C, and 1 mL min^−1^, respectively. The mobile phase comprised methanol and water at a ratio of 98:2 by volume, and an equal elution method was selected. The emission wavelengths 295 and 330 nm were set for fluorescence detection. Finally, *AsA*/*V_E_* was calculated for each group (28).

### 2.9 Glutathione content measurement

Tissue samples (1.5 g) were ground in 2 mL 60 mmol L^−1^ sodium phosphate buffer (pH 7.0), and refrigerated centrifugation was performed at 12,000 × *g* for 15 min, after which 100 μL of supernatant was obtained. Finally, GSH content determined using a Plant Glutathione Elisa kit (FT-P5423Z; Shanghai Fanwei Biotechnology Co., Ltd., Shanghai, China) according to the manufacturer’s instructions (29).

### 2.10 Related enzyme activity determination

A Plant ELISA kit [APX (ml076456), GR, DHAR (ml076454), MDHAR (ml076454); Enzyme-linked Biotechnology Co., Ltd., Shanghai, China], GR activity assay kit (E-BC-K099-S; Ilerite Biotechnology Co., Ltd., Wuhan, China), and *ω*-3 fatty acid desaturase activity assay kit (BLL-Z30245A; Baililai Biotechnology Co., Ltd., Shanghai, China) were used to determine the activity of related enzymes.

### 2.11 Determination of related enzyme gene expression

A MagMAX 96 Total RNA Isolation Kit (Takara Biomedical Technology Co., Ltd., Kyoto, Japan) was used to extract RNA from samples at each sampling point. RNA samples that passed quality inspection were reverse-transcribed into cDNA and used for subsequent gene expression analysis. Gene expression levels were determined using an ABI-Quantstudio DX System (Thermo Fisher Scientific). The relative expression gene levels were calculated using the 2^-ΔΔCt^ method.

### 2.12 Subcellular localization

The BoPPT1 coding region was fused with the pCAMBIA2300-GFP vector and transformed into *Agrobacterium* strain GV3101 (Biovector NTCC Inc., Beijing, China), which was used to infect *Nicotiana benthamiana*; the plants were the cultured for 3 days. Subsequently, a confocal laser scanning microscope (TCS SP8 CARS; Leica Biosystems) was used to observe chlorophyll fluorescence and green fluorescence protein (GFP) fluorescence in the samples at 580 nm and 488 nm, respectively; photos were taken to record subcellular fluorescence morphology (30).

### 2.13 Statistical analysis

Primer 3^1^ was used to design primer sequence (Supplementary Table S1). Data analysis was performed in GraphPad Prism 8 (GraphPad Software, La Jolla, CA, USA) using Student’s *t*-tests. Adobe Illustrator CC 2017 (Adobe Inc., San Jose, CA, USA) was used to construct charts. Protein family BoPPT1 alignment and transport across the membrane structure domain were predicted using InterPro^2^ and Deep TMHMM.^3^

## 3 Results

### 3.1 Changes in color, Chl content, and ultrastructural under different treatment

**Figure 1:**
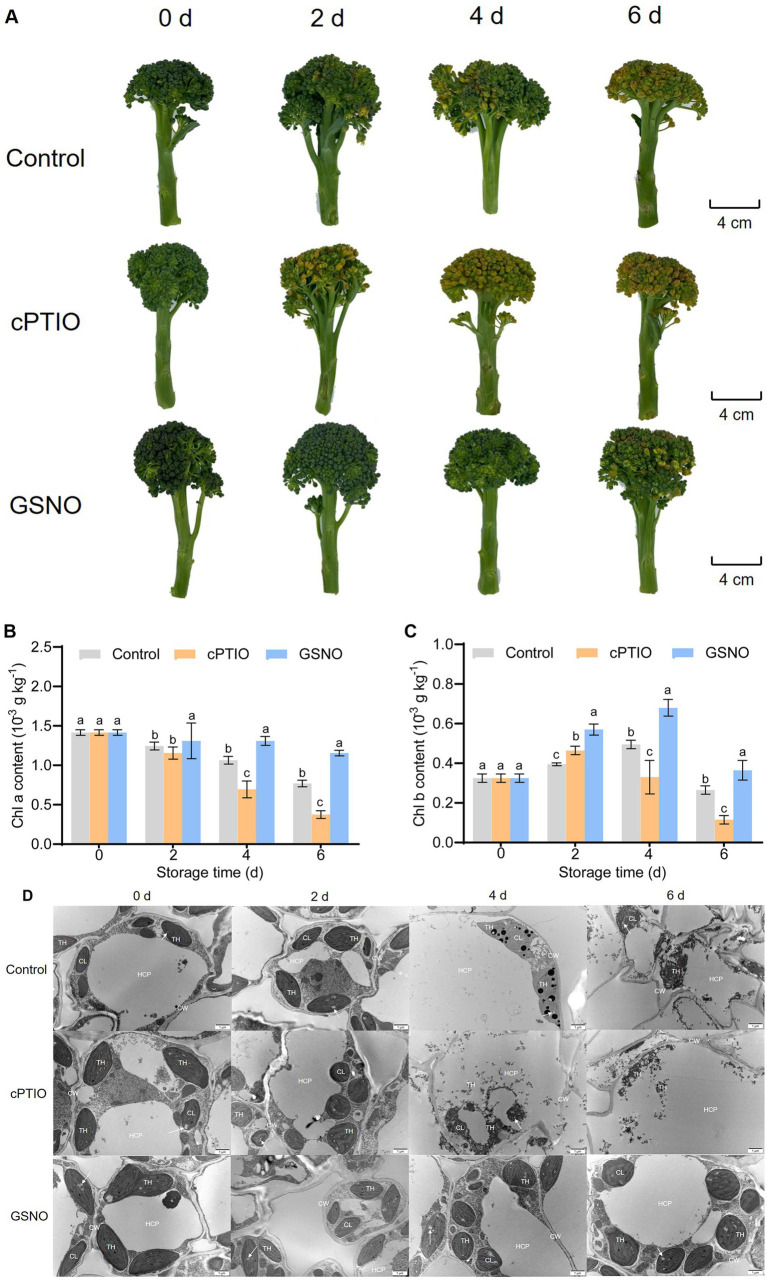
Yellowing-related changes in broccolini under different treatments. **(A)** Phenotype, **(B)** Chl *a* content, **(C)** Chl *b* content, and **(D)** Ultrastructural changes. For cellular structure images, scale bar = 1 μm, magnification = 5,000×. Chl, Chlorophyll; CW, cell wall; CL, chloroplast; TH, thylakoid; HCP, huge-celled parenchyma. The arrow indicates starch.

The control samples showed slight yellowing on day 2, and the cPTIO-treated samples showed moderate to severe yellowing (Figure 1A). The effects of the treatments, based on b*, a*, YI, and Δ*E*, were determined (Table 1): cPTIO treatment resulted in the highest degree of yellowing throughout storage, and the degree of yellowing in the three groups differed significantly on day 4 (*p* < 0.05). On day 4 of storage, yellowing differed between the groups; curd yellowing was delayed in the GSNO treatment. GSNO treatment consistently maintained high Chl *a* and *b* levels (*p* < 0.05) (Figures 1B,C). In the GSNO group, Chl *b* content peaked on day 4. After GSNO treatment, chloroplast morphology was normal, with the chloroplasts located close to the cell wall and having a complete thylakoid membrane system (Figure 1D). In contrast, in the control and cPTIO groups, the chloroplasts were transformed from spindle-shaped to spherical and had migrated toward the cell center, resulting in thinning of their double-layer membrane with extended storage time. The starch within the chloroplasts had significantly expanded and increased, and the thylakoid stromal layer had gradually blurred until it had disintegrated by day 6 in the control group and day 4 in the cPTIO group. These results correspond with the phenotypic findings, indicating that NO can effectively delay postharvest yellowing.

**Table 1:** Changes in color attributes of postharvest broccolini following treatment with an NO donor (GSNO) and an NO scavenger (cPTIO).

Parameters	Treatments	Storage time (*d*)			
		0	2	4	6
b*	Control	5.21 ± 0.04	7.03 ± 0.08^b^	10.22 ± 0.09^b^	13.25 ± 0.11^b^
	cPTIO	5.21 ± 0.04	10.28 ± 0.09^a^	14.44 ± 0.11^a^	16.42 ± 0.12^a^
	GSNO	5.21 ± 0.04	6.65 ± 0.07^b^	8.42 ± 0.06^c^	9.07 ± 0.08^c^
a*	Control	−11.23 ± 0.12	−8.52 ± 0.09^b^	−5.43 ± 0.06^b^	−3.91 ± 0.04^b^
	cPTIO	−11.23 ± 0.12	−5.11 ± 0.08^a^	−2.12 ± 0.03^a^	−1.42 ± 0.08^a^
	GSNO	−11.23 ± 0.12	−9.26 ± 0.07^b^	−8.28 ± 0.10^c^	−10.06 ± 0.13^c^
YI	Control	31.01 ± 0.28	37.03 ± 0.33^b^	52.41 ± 0.43^b^	79.64 ± 0.78^b^
	cPTIO	31.01 ± 0.28	58.74 ± 0.42^a^	65.82 ± 0.35^a^	92.31 ± 0.63^a^
	GSNO	31.01 ± 0.28	35.89 ± 0.21^b^	40.09 ± 0.23^c^	51.83 ± 0.44^c^
Δ*E*	Control	4.75 ± 0.21^b^, large difference			
	cPTIO	6.03 ± 0.37^a^, very large difference			
	GSNO	1.23 ± 0.15^c^, slight difference			

b*: yellowness; a*: greenness; YI: yellowing index; Δ*E*: color difference. Δ*E* was categorized into standard size classes (30): <0.05 (no difference), 0.5–1.5 (slight difference), 1.5–3.0 (moderate difference), 3.0–6.0 (large difference), and >6.0 (very large difference). Data were obtained using three biological replicates. Differences with *p* < 0.05 were considered significant. ^a,b,c^Different letters in different columns represent differences between samples (*p* < 0.05).

### 3.2 Indicators of membrane damage changes during broccolini yellowing

**Figure 2:**
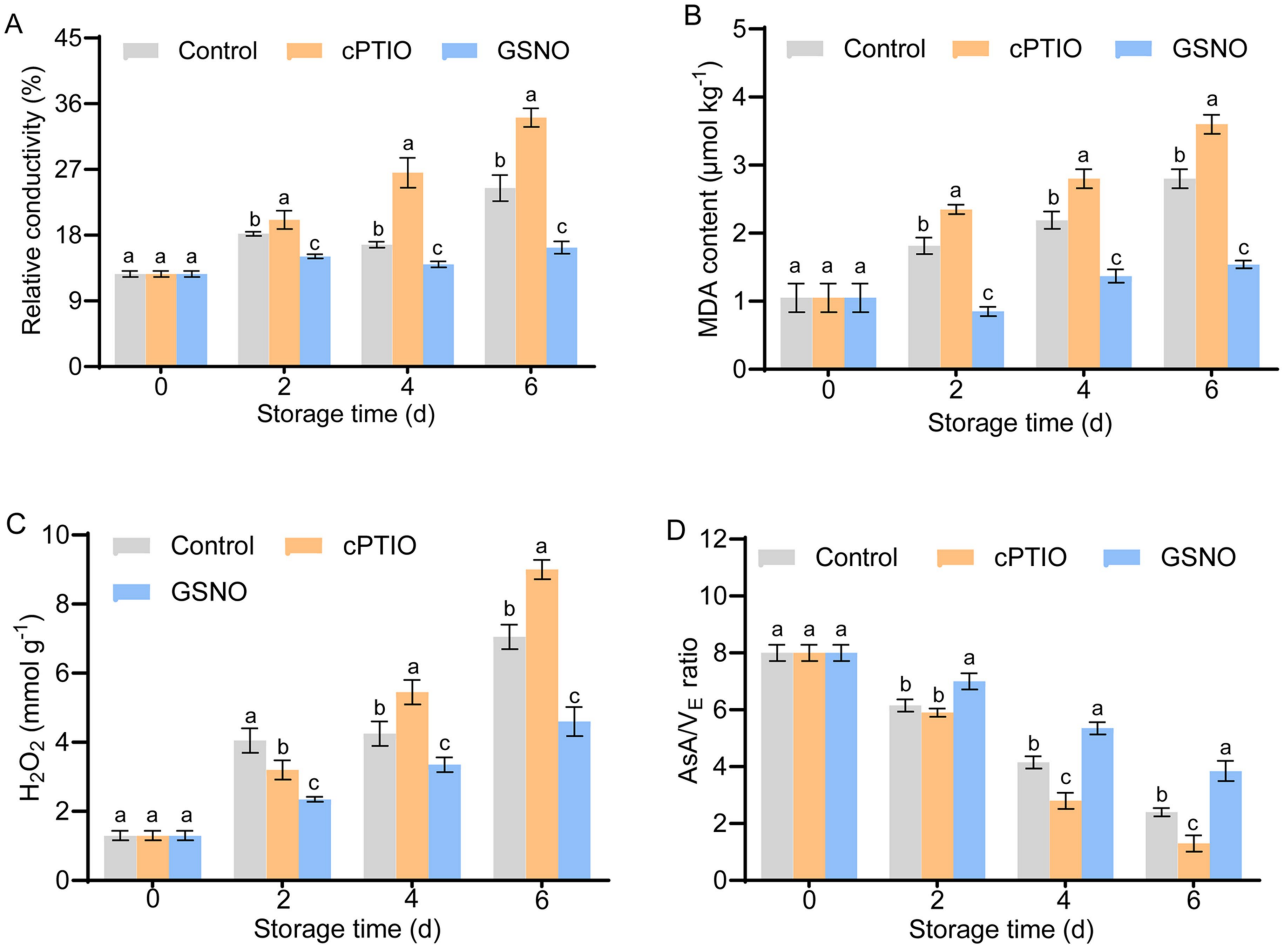
Membrane damage indicating changes in broccolini under different treatments. Changes in **(A)** relative conductivity, **(B)** MDA content, **(C)** H_2_O_2_ content, and **(D)** *AsA*/*V_E_* ratio. Values are means ± standard deviation (*n* = 9). Different letters identify groups that differ significantly (*p* < 0.05) based on Student’s *t*-tests. MDA, malondialdehyde; AsA, ascorbic acid.

In the control and GSNO groups, relative conductivity fluctuated and increased throughout storage; it increased sharply in the cPTIO samples, which had significantly higher relative conductivity than the other two groups (*p* < 0.05) (Figure 2A). MDA content in the GSNO group decreased temporarily on day 2 of storage; in the control and cPTIO groups, however, it increased significantly, and by the end of the storage period was 46 and 61% higher, respectively, than in the GSNO group (Figure 2B). Throughout storage, the H_2_O_2_ content of each group increased continuously; relative to the initial levels (day 0), it was 3.5-, 4.6-, and 2.3-fold higher, respectively, in the control, cPTIO, and GSNO groups by the end of the storage period (Figure 2C). The *AsA*/*V_E_* ratio decreased with storage time; at the end of the storage period, it was higher in the GSNO treatment than in the other two groups (Figure 2D). These results indicate that NO effectively maintained antioxidant system the efficiency and alleviated oxidation-induced membrane damage.

### 3.3 Changes in membrane lipid components and fatty acid component during broccolini yellowing

**Figure 3:**
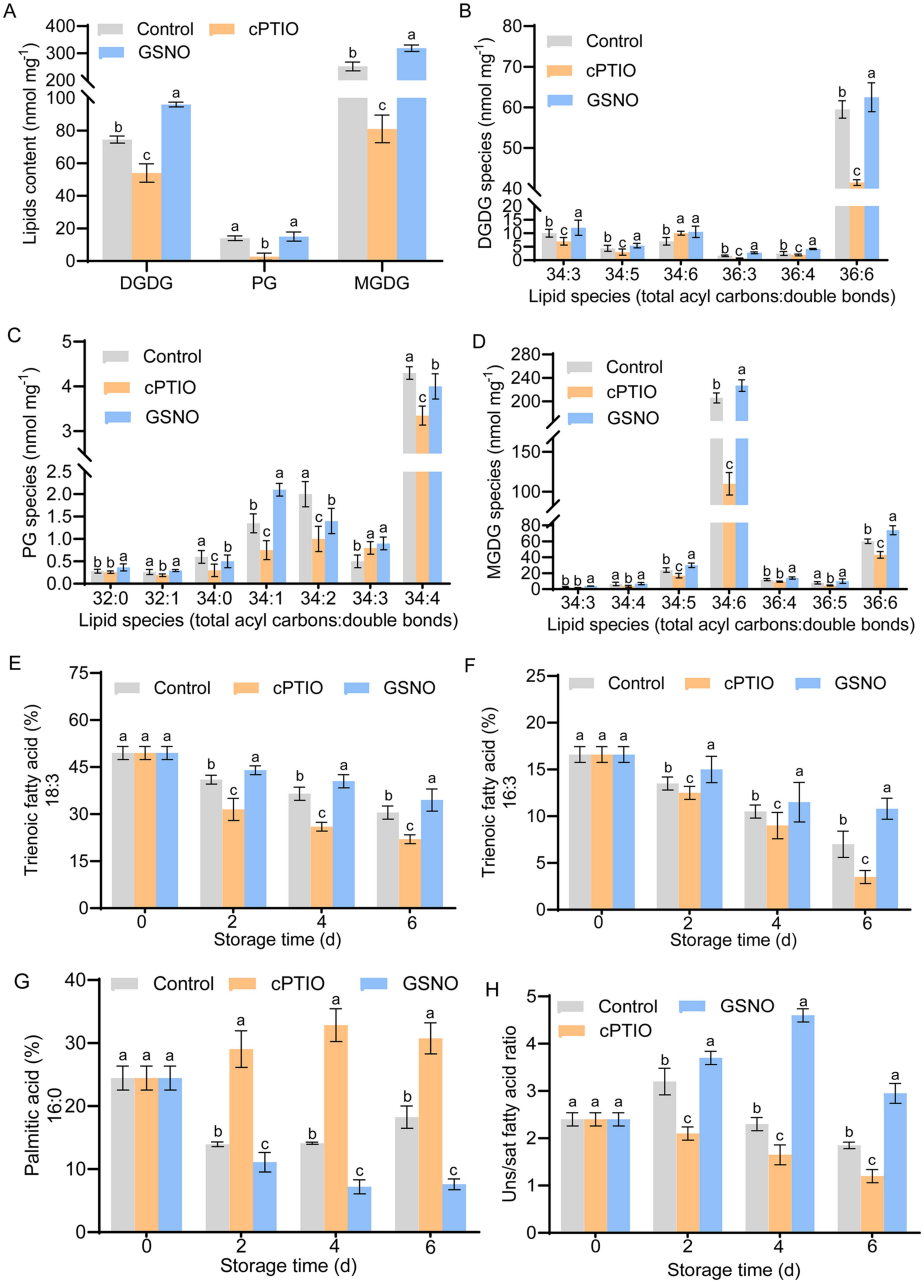
Changes in membrane lipid and fatty acid component levels in broccolini under different treatments. Changes in **(A)** total lipid and **(B)** DGDG, **(C)** PG, and **(D)** MGDG species content in broccolini on day 4 postharvest under different treatments. Changes in the fatty acid composition in broccolini during storage: **(E)** 18:3-trienoic fatty acid, **(F)** 16:3-trienoic fatty acid, **(G)** 16:0-palmitic acid, and **(H)** unsaturated-to-saturated fatty acid ratio. Values are means ± standard deviation (*n* = 9). Different letters identify groups that differ significantly (*p* < 0.05) based on Student’s *t*-tests.

Total lipids were measured on day 4, when yellowing was most severe. The main components of the chloroplast membrane, including monoglycosyldiacylglycerol (MGDG), phosphatidylglycerol (PG), and digalactosyldiacylglycerol (DGDG), were analyzed (31). PG content was 82% lower in the cPTIO group than in the control; however, it did not differ significantly between the GSNO and control groups (Figure 3A). Levels of DGDG and MGDG, two key fatty acids, were significantly higher in the GSNO group than in the control and cPTIO groups, and were lowest in the cPTIO group (*p* < 0.05). Six DGDG species were detected, and (except for 34:6-DGDG) their content was significantly higher in the GSNO group than in the other two groups (Figures 3B–D). The 34:6-DGDG content did not differ significantly between the cPTIO and GSNO groups, but was 25% higher in these groups than in the control (*p* < 0.05). Relative to the GSNO group, the 32:0-PG content of the cPTIO group was 18% lower, and did not differ significantly from that of the control. Relative to the cPTIO group, the 32:1-PG content of the GSNO group was 23% higher, and did not differ significantly from that of the control. The levels of 34:0-, 34:2-, and 34:4-PG were significantly higher in the control than in the other groups. Relative to the control, the 34:3-PG content of the GSNO-treated group was 34% higher, and did not differ significantly from that of the cPTIO group. The 34:1-PG content of the GSNO group was significantly higher than that of the other two groups (*p* < 0.05). Among the seven MGDG species, 34:6-MGDG exhibited the highest content. The content of 34:4- and 36:5-MGDG was 40% higher in the control and GSNO groups than in the cPTIO group (*p* < 0.05).

The levels of both types of triene fatty acids (18:3, 16:3) declined during storage, but were consistently significantly higher in the GSNO group than in the other groups (Figures 3E,F); their levels decreased the most in the cPTIO group. In the control and cPTIO groups, the proportion of 16:0 binary acid fluctuated and increased throughout storage, whereas it decreased continuously in the GSNO group. The ratio of unsaturated to saturated fatty acids was much higher in the GSNO group than in the control and cPTIO groups, and peaked on day 4 in the GSNO group (Figures 3G,H). In summary, GSNO treatment delayed the sharp reduction in unsaturated fatty acid levels during storage and maintained postharvest chloroplast membrane lipid levels.

### 3.4 Changes in membrane lipid metabolism-related enzymatic activity and gene expression during broccolini yellowing

**Figure 4:**
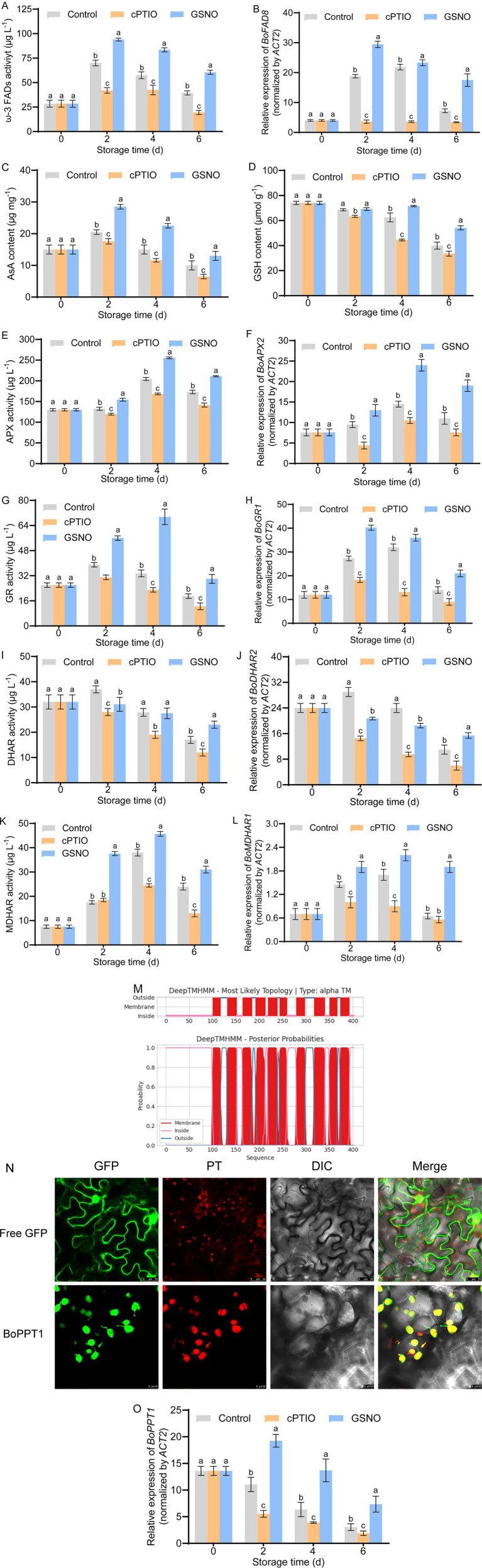
Changes in enzymes and genes related to lipid membrane metabolism and in antioxidant-related indicators in broccolini during storage under different treatments; characterization of BoPPT1. Activity of **(A)** *ω*-3 FADs, **(E)** APX, **(G)** GR, **(I)** DHAR, and **(K)** MDHAR enzymes. Content of **(C)** AsA and **(D)** GSH. Expression of **(B)** *BoFAD8*, **(F)** *BoAPX2*, **(H)** *BoGR1*, **(J)** *BoDHAR2*, **(L)** *BoMDHAR1*, and **(O)** *BoPPT1*. **(M)** Prediction of transmembrane transport domains of BoPPT1. Red: TM-helix transmembrane protein region; Blue: membrane structure; Pink: outer membrane. **(N)** Subcellular localization of BoPPT1 in *Nicotiana benthamiana.* GFP represents the green fluorescence field, PT represents the chlorophyllin marker field (mCherry), DIC indicates the field, Merge represents the superposition field. Values are means ± standard deviation (*n* = 9). Different lowercase letters identify groups that differ significantly (*p* < 0.05) based on Student’s *t*-tests.

In the unsaturated fatty acid synthesis pathway, *ω*-3 fatty acid desaturase (FAD) catalyzes and synthesizes the 18:3 and 16:3 types of TAs (32). The ω-3 FAD enzyme activity in both the control and GSNO treatment group showed fluctuating changes during storage, and both reached their peaks on day 2. ω-3 FAD enzyme activity was lowest in the cPTIO group (Figure 4A). The expression of *BoFAD8* (Figure 4B) changed similarly to ω-3 FAD enzyme activity: on day 6, *BoFAD8* expression was 56 and 72% higher, respectively, in the GSNO group than in the control and cPTIO groups, respectively (*p* < 0.05). This indicates that GSNO treatment contributed to maintaining ω-3 FAD enzyme activity and *BoFAD8* transcription levels during postharvest storage.

### 3.5 AsA–GSH cycle level changes in broccolini under different treatment

AsA content varied significantly between the treatments (Figure 4C). On day 2 of storage, the AsA content of the GSNO group peaked at 28.1 μg mg^−1^, then decreased; on day 6, it was 47 and 60% higher than in the control and cPTIO samples, respectively. GSH content varied slightly among the groups during the first 2 days of storage, differing significantly later in storage; it remained highest in the GSNO group (*p* < 0.05) (Figure 4D). DHAR, GR, MDHAR, and APX, key enzymes in the AsA–GSH cycle, exhibited protein and gene transcriptional levels that were consistent (Figures 4E–L). Their levels were significantly higher in the GSNO group than in the other two groups and were lowest in the cPTIO group (*p* < 0.05).

### 3.6 *BoPPT1* gene expression and bioinformatics analysis during broccolini yellowing

The protein sequence of BoPPT1 was compared with those of AtPPT1 (from *Arabidopsis thaliana*, NP_19831.7), BnPPT1 (from *Brassica napus*, NP_00130291.1), and BrPPT1 (from *Brassica rapa*, XP_009108071.1). The BoPPT1 sequence was >97.07% similar to that reported for PPT1, and this protein was thus preliminarily confirmed as PPT1 (Supplementary Figure S1). Based on the bioinformatics results (Figure 4M), six transmembrane (TM)-helix domains were predicted within amino acids 101–390 of BoPPT1 (102–119, 129–151, 164–186, 219–241, 276–298, and 371–390); BoPPT1 thus acts as a membrane-binding protein for embedding into the cell membrane. These results imply that BoPPT1 performs transmembrane protein transport.

Based on subcellular localization, BoPPT1 was localized in chloroplasts (Figure 4N). During storage, *BoPPT1* transcription levels were consistently 2–3-fold higher in the GSNO group than in the control and cPTIO groups (*p* < 0.05), indicating that GSNO treatment can effectively delay fatty acid changes in broccolini chloroplast membranes (Figure 4O). These results indicate that the protein encoded by *BoPPT1* is a chloroplast-membrane spanning protein that further regulates postharvest yellowing of broccolini under the influence of exogenous NO.

## 4 Discussion

Our findings support the hypothesis that NO treatment improves antioxidant protection ability and regulates chloroplast membrane metabolism, thus improving postharvest storage quality in broccolini. Harvesting and storage cause extensive production of ROS on subcellular surfaces in fruits and vegetables, owing to high levels of respiration. ROS decompose unsaturated fatty acids in biofilm phospholipids, inducing membrane-structure breakdown, altering biofilm permeability, and leading to pigment destruction. The AsA–GSH cycle includes important substrates that function as non-enzymatic antioxidants that can effectively clear ROS, maintain cell homeostasis, and resist cell damage. The chloroplasts of higher plants contain two APX isoenzymes, sAPX and tAPX, located in the chloroplast matrix and on the thylakoid membrane, respectively. APX decomposes H_2_O_2_ generated during AsA–GSH cycle by catalyzing the reaction between AsA and H_2_O_2_. Similarly, GR can effectively scavenge ROS such as H_2_O_2_ under oxidative stress, and can keep GSH levels low, thus protecting against biofilm damage caused by lipid peroxidation. Under DHAR catalysis, dehydroascorbic acid combines with GSH, reducing it to AsA. AsA accumulation protects the biofilm system against oxidative damage, thereby delaying loss of quality, and MDHAR can regenerate AsA, thereby improving antioxidant levels in biofilm systems (15). Here, H_2_O_2_ levels were significantly elevated in the control and cPTIO groups, with a sharp decline in MDHAR, APX, GR, and DHAR enzyme activity and transcriptional levels. GSNO treatment effectively reduced relative conductivity and MDA content, helping to improve antioxidant system efficiency. Comparable results have been reported for peaches, in which exogenous NO effectively regulated membrane metabolism peroxidation, thereby alleviating chilling damage (33). Therefore, in broccolini, NO enhances ROS scavenging by enhancing the AsA–GSH cycle, thereby maintaining the chloroplast biofilm system and delaying postharvest yellowing.

The phosphoenolpyruvate/phosphate translocator (PPT) assists in transporting phosphoenolpyruvate between the cytoplasm and chloroplasts, contributing to the maintenance of chloroplast morphology and affecting membrane metabolism. After *AtPPT1*-knockout, chloroplast morphology in *Arabidopsis thaliana* was abnormal, and the leaves were significantly yellowed compared to those of the wild-type (34), with similar findings for *Brassica napus* L. (35). Here, the GSNO group exhibited higher Chl *a* and *b* content and *BoPPT1* transcription than the other groups, and chloroplast morphology showed no significant change with prolonged storage in this group. PPT proteins are one of three types in the triose-phosphate transporter (TPT) family; these transporters typically have 4–10 transmembrane helices (36). Here, we found that BoPPT1 was localized in chloroplasts and contained six TM-helix domains, consistent with the characteristics of PPT proteins. Therefore, we predict that BoPPT1 is a chloroplast membrane shuttle protein that can regulate chloroplast morphology and further broccolini yellowing.

The GSNO group exhibited high levels of the main membrane components, DGDG, PG, and MGDG. The changes in in membrane component levels during storage were consistent with those in Chl content. PG, DGDG, and MGDG are relatively abundant chloroplast membrane components in cruciferous plants. The thylakoid membrane is a highly dynamic lipid matrix that resists environmental changes. PG, a key chloroplast membrane component, cooperates with hydrophobic metabolites to shuttle between the thylakoid membranes (37). PG molecules are surrounded by a semi-creamy lipid monolayer and are directly connected to the extracapsular lumens of thylakoids, thereby coregulating hydrophobic metabolite shuttling between the thylakoid membranes (38). PG thus links morphological changes in the thylakoid membranes to the stress response. In rice (*Oryza sativa* L. subsp. *japonica*), *OsFBN7* overexpression significantly increases PG content and contributes to maintaining normal chloroplast morphology and function (39).

DGDG and MGDG are lipid components of bi- and monolayer membranes, respectively, and the DGD:MGDG ratio is an important indicator of membrane integrity. They are actively involved in regulating chloroplast thylakoid membrane fluidity and integrity, and participate actively in improving plant resistance to stress (40). Loss of quality in fruits and vegetables during postharvest storage is a manifestation of stress. In tobacco (*Nicotiana tabacum* L.) under stress, MGDG synthetase gene overexpression increased 36:4- and 36:6-DGDG and MGDG species levels, thus damaging chloroplast membrane function and structure (41). Chloroplast membrane transport proteins are involved in the synthesis and transport of chloroplast membrane lipids and are related to the integrity of the chloroplast membrane system and the homeostasis of thylakoid stacking structure, which leads to their key role in regulating chloroplast morphology and function. Ultimately, the change in chloroplast morphology directly affects the yellowing process of plants (42). Therefore, we speculate that *BoPPT1* may affect chloroplasts ultrastructure and participate in the synthesis of chloroplast precursor substances and Chl, thereby delaying postharvest yellowing in broccolini.

The non-enzymatic antioxidant scavenging system generates oxygen free radicals that attack polyunsaturated fatty acids in biofilms, triggering lipid peroxidation and affecting the ratio of unsaturated to saturated fatty acids. As the main components of unsaturated fatty acids in chloroplast membranes, thylakoid lipids exhibit the highest degree of unsaturation. In terms of their fatty acids, the inner and outer membranes of chloroplasts are highly unsaturated, and 70% of their fatty acids comprise 18:3 (*α*-linolenic)-type and 16:3 (hexadecatrienoic acid)-type TAs. The main component of saturated fatty acids is 16:0 palmitic acid (43). *α*-Linolenic and hexadecyclic acids each have three double bonds, and are 18:3- and 16:3-type TAs, respectively; together, they account for 90% of the fatty acids of monosaccharide glycerol and >60% of the fatty acid composition of the thylakoid membrane. Fatty acid desaturation is an important process in plant stress resistance. Saturated fatty acids can form polyunsaturated fatty acids or monounsaturated fatty acids under the action of desaturases.

Here, the main saturated fatty acid in broccolini chloroplasts was palmitic acid, accounting for approximately 20% of its fatty acid content. Monounsaturated fatty acids can also form polyunsaturated fatty acids. In chloroplasts, for example, *ω*-3 FAD catalyzes the formation of double bonds in the acyl chains of oleic acid, thus transforming it into α-linolenic acid. ω-3 FADs are key enzymes in the synthesis of unsaturated fatty acids and play a crucial role in regulating biofilm formation and plant stress resistance. In contrast, α-linolenic acid, an important precursor of cellular jasmonic acid (JA) signaling molecules after α-linolenic acid separation from plant cell membranes, results in the accumulation of large amounts of JA via the catalytic action of various enzymes. JA activates antioxidant enzyme activity, improving ROS scavenging ability (44). The JA signaling pathway can interact with multiple signaling pathways to maintain plant stress levels. Exogenous NO-induced strong expression of alkenoxide synthase and lipoxygenase genes in *A. thaliana*, altering JA signal transduction levels and effectively improving stress resistance (45). Here, during storage, the GSNO group exhibited significantly higher levels of ω-3 FAD enzyme activity and *BoFAD8* transcription than the control and cPTIO groups. This indicates that exogenous NO mediates JA signal transduction and increases *ω*-3 FAD enzyme activity, thereby regulating chloroplast biofilm components and ensuring a high ratio of unsaturated to saturated fatty acids. This in turn maintains chloroplast inner and outer membrane morphology, delaying the postharvest yellowing of broccolini. This view is a speculation based on our results. In subsequent studies, we will confirm the role of this pathway in broccolini through quantitative detection of endogenous JA and expression levels of JA-specific biosynthetic enzymes.

## 5 Conclusion

**Figure 5:**
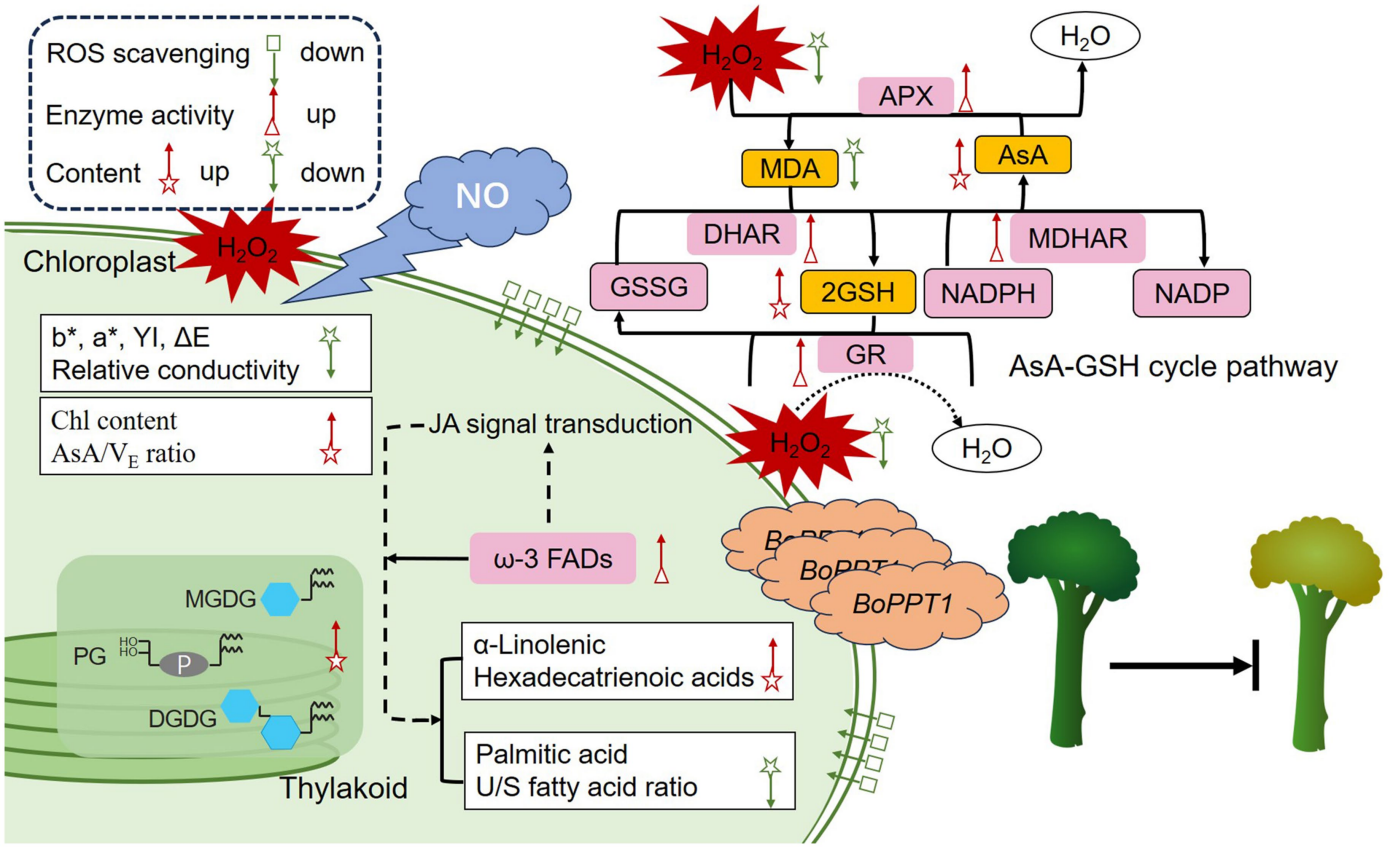
Regulatory model of the NO-mediated mechanisms delaying postharvest yellowing of broccolini.

These findings are summarized in the regulatory model presented in Figure 5. Adjusting the concentration of GSNO to 80 μM effectively delayed the yellowing of broccolini beyond day 4, whereas yellowing occurred earlier in the control. GSNO treatment-maintained chloroplast membrane morphology and good thylakoid membrane development, reduced relative conductivity and MDA and H_2_O_2_ content, and increased the *AsA*/*V_E_* ratio, thus maintaining chloroplast biofilm system stability. Levels of key components of chloroplast membrane lipids were highly elevated in the GSNO group. GSNO improved ω-3 FAD enzyme activity and *BoFAD8* transcription, thus increasing unsaturated fatty acid content and the ratio of unsaturated-to-saturated fatty acids in chloroplasts. GSNO treatment enhanced AsA–GSH antioxidant system activity by increasing GR, DHAR, MDHAR, and APX enzyme activity, AsA and GSH content, and upregulated *BoAPX2*, *BoGR1*, *BoDHAR2*, and *BoMDHAR1* transcription; it also upregulated *BoPPT1*, which is critical in maintaining fatty acid components and related metabolism. *BoPPT1*, a transmembrane transport protein, can shuttle through chloroplasts and the inner and outer cell membranes, thereby regulating postharvest yellowing.

These findings elucidate the regulatory mechanisms of exogenous NO on the postharvest yellowing of broccolini in terms of chloroplast membrane lipid metabolism and AsA–GSH cycle antioxidant activity. These findings provide a theoretical basis for extending postharvest storage quality of vegetables. Further experimental verification is needed to investigate the complex molecular mechanisms underlying the effects of exogenous NO on the stability of the chloroplast membrane and the antioxidant system in postharvest broccolini.

## Data Availability

The original contributions presented in the study are included in the article/Supplementary material, further inquiries can be directed to the corresponding author.

## Footnotes

1
http://frodo.wi.mit.edu/

2 http://www.ebi.ac.uk/interpro

3
https://dtu.biolib.com/DeepTMHMM
