# Supplementary material for: Nitric oxide delays yellowing by regulating chloroplast membrane lipid metabolism and the ascorbate–glutathione cycle in postharvest broccolini
Source: Front Nutr. 2026 Feb 23;13:1785583. doi: 10.3389/fnut.2026.1785583 (PMC12968298; doi:10.3389/fnut.2026.1785583)
Supplement: Supplementary file 1 [file Supplementary_file_1.docx]

Supplementary Material

# Supplementary Tables

# Table 1 List of primers used in this study

| Primer | Sequence (5’-3’) | |
| --- | --- | --- |
|  | Forward | Reverse |
| **Gene expression** | | |
| *Act2* | CCGAGAGAGGTTACATGTTCACCAC | GCTGTGATCTCTTTGCTCATACGGTC |
| *BoFAD8* | GCCTCTAACCCATCACCCA | GCCACATTCAACGCCCAGT |
| *BoAPX2* | CAGTGAGCGAAGATTACCA | CAAATGTTCCAGCAGAGTG |
| *BoGR1* | GGCAGAAATCCTAATACC | TACAGCACAGGCTACAT |
| *BoPAL* | GGCTTCGTATTGCTCTG | CTTTCTTCGCCACTTGA |
| *BoDHAR2* | CGAGTTAGAAGCCCTGGAA | AGAACAAAGCGTTAGCGTA |
| *BoMDHAR1* | TGAACCAGCAAGTAACAA | CACCAGGACCACCACAT |
| *BoPPT1* | AATAATGTGGCTCCTC | TTGATTGTATGGGTGA |
| **Subcellular localization** | | |
| *pCAMBIA2300-GFP*-BoPPT1 | cgc**GGTACC**AGCATGCAACCAC | cgg**CATATG**GAGGCGTGTGGCCAGT |

The underlined represents the cleavage sites. Lowercase sequence represents the homologous gene sequence of the vector.

# Supplementary Figures


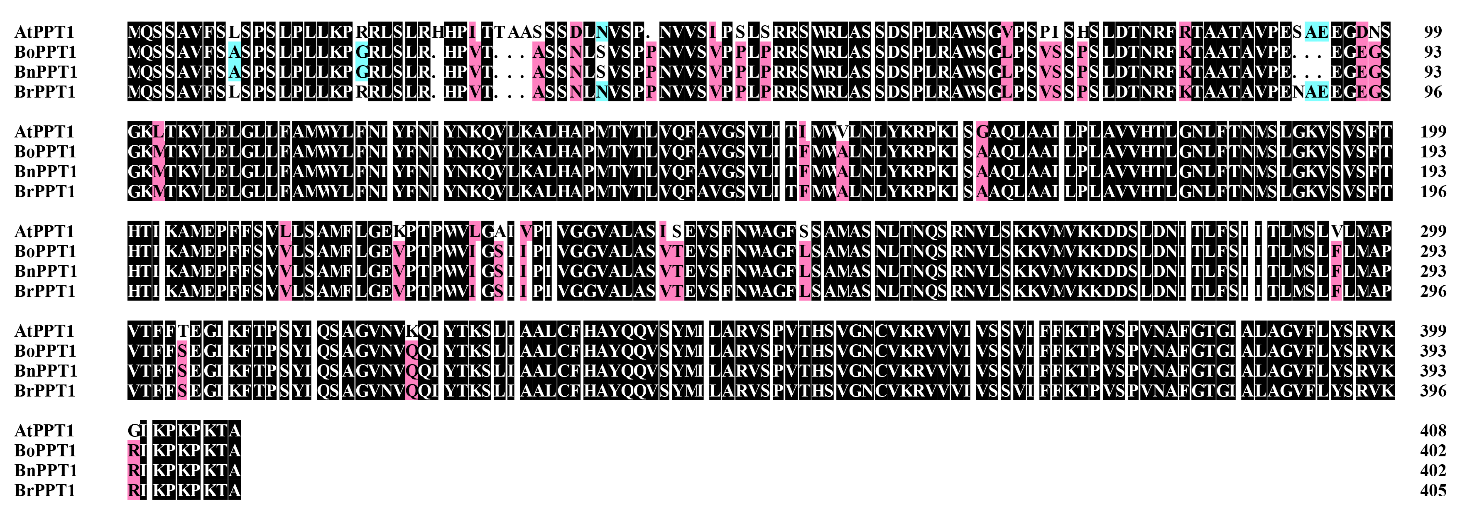
**Figure 1** Multiple alignment analysis *BoPPT1* from other species. The amino acids with an identity of 100% are shown in black; greater than 75% identity shown in pink; between 50% and 75% identity shown in cyan. Dotted lines indicate gaps.
